# Symptomatic Primary Hyperparathyroidism as a Risk Factor for Differentiated Thyroid Cancer

**DOI:** 10.1155/2018/9461079

**Published:** 2018-11-18

**Authors:** Guadalupe Vargas-Ortega, Lourdes Balcázar-Hernández, Baldomero González-Virla, Claudia Ramírez-Rentería, Oriana Nieto-Guzmán, Ana Pamela Garrido-Mendoza, Marco Antonio Flores-Maya, Moisés Mercado, Mendoza-Zubieta Victoria

**Affiliations:** ^1^Endocrinology Department, Hospital de Especialidades, Centro Médico Nacional Siglo XXI, Instituto Mexicano del Seguro Social, Cuauhtemoc 330, Colonia Doctores, 06720 México City, Mexico; ^2^Experimental Endocrinology Unit, Hospital de Especialidades, Centro Médico Nacional Siglo XXI, Instituto Mexicano del Seguro Social, Cuauhtemoc 330, Colonia Doctores, 06720 México City, Mexico

## Abstract

**Background:**

The primary hyperparathyroidism (PHPT) is a common disease for the endocrinologist. The concomitant thyroid disease and differentiated thyroid cancer (DTC) appear to be more frequent in patients with PHPT than in the general population. The aim of this study was to characterize patients with symptomatic PHPT with and without DTC and analyze frequency and risk factors.

**Methods:**

We consecutively studied patients with symptomatic PHPT diagnosed and treated at our center between 2013 and 2015. Patients with subclinical and syndromic forms of PHPT were excluded. Clinical and biochemical characteristics of patients with and without DTC were compared and risk factors were determined. All patients were studied with thyroid ultrasound and thyroid gammagraphy with TC-MIBI. Two expert surgeons performed all the surgical procedures.

**Results:**

In 59 patients included, we found 12 cases of PTC (20.3%). The final histopathological report of the PTC was 7 cases of follicular variant, 2 cases of oncocytic variant, 2 cases of classic variant, and 1 case of columnar cells variant of PTC. Patients with thyroid cancer were older than patients without thyroid cancer (62 ± 9.5 versus 52 ± 15.8, p = 0.03). Higher preoperative levels of iPTH were associated with PTC (p=0.03) [OR 5.16 (95% CI: 1.08-24.7)].

**Conclusion:**

PTC is frequent in patients with symptomatic PHPT. Thyroid nodules in patients with symptomatic PHPT must be studied before parathyroidectomy. In symptomatic PHPT, higher level concentration of parathormone (PTH) was associated with higher risk of DTC.

## 1. Introduction

Primary hyperparathyroidism (PHPT) is the principal cause of hypercalcemia in ambulatory patients and is one of the most common endocrine diseases [1–3]. It is more frequent in women and it increases with age. Subclinical or asymptomatic presentation is the most common in occidental countries like United States and Europe and the diagnosis is often made incidentally [2–5].

Prospective follow-up studies of asymptomatic PHPT have shown that it is a progressive disease; after 10 to 15 years, up to one-third of the subjects showed a progression with evident characteristics of the disease, for example, kidney stones, worsening of hypercalcemia, and reduction of bone mineral density (BMD) [6].

The natural history of PHPT is unknown. It is highly probable that patients with clinical PHPT have a longer evolution with constant exposition to high calcium levels and parathormone (PTH) with a progressive damaging effect in the principal target organs like bone and kidney [1, 2]. In observational studies, the severity and chronicity of PHPT have been associated with a higher cardiovascular morbimortality and multiple neoplasms; this association with asymptomatic PHPT has not been found [5, 7, 8].

About 15-75% of patients with PHPT may have coexistent thyroid disease, including benign and malign nodules [9]. The prevalence of differentiated thyroid cancer (DTC) in this context appears to be higher than general population; the prevalence found goes from 2 to 28% [9, 10]. The common risk factor for thyroid and parathyroid neoplasms is the history of neck radiation, yet this factor is not always present [11].

The objective of this study is to characterize patients with symptomatic primary hyperparathyroidism with or without differentiated thyroid cancer.

## 2. Patients and Methods

Patients with biochemically documented PHPT diagnosed and treated at our center between 2013 and 2015 were included in this study. Patients with subclinical PHPT and patients with HPT associated with hereditary syndromes were excluded. The study protocol was approving by our local ethics and scientific committees and all patients signed the appropriate informed consent. All patients were evaluated by a preoperative ^99m^Tc-sestamibi scintigraphy (^99m^Tc-MIBI) as well as high-resolution neck ultrasonography (USG).

### 2.1. Biochemical and Hormonal Measurements

Biochemical evaluation was performed on an Architect-ci8200 automated analyzer (Abbott Diagnostics, Abbott Park, Illinois) and included serum glucose, albumin, total cholesterol, high-density lipoprotein- (HDL-) cholesterol, low-density lipoprotein- (LDL-) cholesterol, triglycerides, total calcium, magnesium, and phosphorus. We used a specific chemiluminescent assay to measure PTH intact molecule (DiaSorin Inc., Stillwater, Minesota) with a sensitivity of 1 pg/mL, inter- and intra-assay coefficients of variation (CV) of 5.3% and 3.5%, respectively, and a normal reference range of 15-65 pg/mL). Serum 25 (OH) D3 was measured by means of a chemiluminescent assay (DiaSorin Inc., Stillwater, Minnesota) with a sensitivity of 4 ng/ml and inter- and intra-assay CVs of 5.1% and 8.6%, respectively.

### 2.2. Neck Ultrasonography and Parathyroid Scintigraphy Protocols

Neck USG was performed using Aixplorer Ultramedics, scanner with an 8-14 MHz transducer. Ultrasound findings were categorized according to the TIRADS system [12]. Parathyroid scintigraphy was carried out after the intravenous injection of 450 MBq ^99m^Tc-MIBI sestamibi (methoxy-isobutyl-isonitrile). Planar images were acquired using a low-energy, high-resolution collimator positioned as near to the neck as possible using a single-head Siemens E-Cam gamma camera. The early images were acquired in the anterior, left, and right anterior 30° oblique view 15 min after tracer administration (thyroidal phase). Similar views were obtained starting 90-120 min after injection (delayed images).

### 2.3. Surgical Procedures

Patients with a localized parathyroid lesion and without thyroid abnormalities on neck US underwent minimally invasive resection of the parathyroid adenoma. Bilateral neck exploration was performed when the ^99m^Tc-MIBI showed either uptake in more than one parathyroid gland or no uptake at all, and/or the neck US revealed the presence of one or more thyroid nodules larger than 1 cm. Patients with nodules smaller than 1 cm but with a sonographic pattern suggestive of malignancy (hypoechoic, taller-than-wide shape, being with microcalcifications, and ill-defined borders) or with risk factors [12, 13] underwent preoperative ultrasound-guided fine needle aspiration (FNA) biopsy. When the thyroid cytology [14] was clearly benign (Bethesda II) and the parathyroid scan confirmed the presence of a single and distinct parathyroid lesion, the patient was subjected to a minimally invasive parathyroidectomy. The bilateral or classical neck exploration was made when 99mTc-MIBI showed uptake in more than one parathyroid gland, discordant, or nonlocalizing preoperative imaging (suggestive data of parathyroid hyperplasia).

Those patients with nodules larger than 1 cm whose thyroid cytology was classified as Bethesda III, IV, V, or VI were submitted neck exploration and the surgical strategy (lobectomy with or without isthmusectomy, partial thyroidectomy, and total or nearly total) was decided in function of an analysis of risk factors, ultrasound malignity suspicion, multifocality or suspicious adenopathies by ultrasound, and thyroid nodule localization [13]. In our center, preoperative molecular markers determination is not made in a routinary way in patients with indeterminate FNA cytology. Two expert surgeons performed all the surgical procedures.

### 2.4. Statistical Analysis

Quantitative variables are described as means with standard deviation (SD) or medians with interquartile range (IQR) according to their distribution. Proportions (expected frequency, prevalence) were used for the qualitative variables. To establish associations between the continuous quantitative variables we used Student's t-test, the Mann-Whitney U-test, or the Wilcoxon test. For qualitative variables we used the X^2^ or Fisher test, according to the expected value in the boxes. A multivariate, stepwise logistic regression analysis was carried out to explore which clinical and biochemical characteristics were associated with PCT. Cox proportional hazard regression model was used for competing risks. A p<0.05 was considered as statistically significant. Statistical software consisted of SPSS version 17 and STATA version 11.

## 3. Results

A total of 59 patients were included in the study. The mean age was 54.1±15 years and the 77% (n=46) were women. The beginning of biochemical study for PHPT was the evaluation of nephrolithiasis in 28 patients (47.4%), osteoporosis by dual energy X-ray absorptiometry (DEXA) in 29 patients (49%) and 2 patients (3.4%) that presented acute pancreatitis. The diagnosis of PHPT was established with the biochemical profile. Target organ damage was evaluated in all patients. The baseline demographic, clinical, biochemical, and histopathological characteristics were described in Table 1.

In the neck ultrasound, normal thyroid was found in 27 patients (45%), thyroiditis (diffuse echogenicity without distinct nodular lesions) in 4 patients (7%), multinodular goiter in 8 patients (14 %), and solitary thyroid nodule in 20 patients (34 %).

In patients with multinodular goiter, the FNA biopsy of the suspected nodule reported papillary thyroid cancer (preoperative diagnosis) in 4 cases. Near-total thyroidectomy was performed in patients with multinodular goiter and PHPT. In patients with a single thyroid nodule <1 cm, the FNA biopsy reported papillary thyroid cancer (preoperative diagnosis) in 2 cases. In the rest of the patients with nodules> 1 cm, in 2 cases the FNA biopsy reported Bethesda VI. Of the nodules with Bethesda III, IV, and V, in 4 cases the CPT was corroborated in the histopathological study.

The final histopathologic report confirmed the diagnosis in all cases. The final histopathological diagnosis of PHPT was adenoma in 51 patients (86%) and hyperplasia in 8 patients (14%).

A total of 12 patients (20.3%) presented papillary thyroid cancer (PTC) concomitantly. The final histopathological report of the PTC was 7 cases of follicular variant, 2 cases of oncocytic variant, 2 cases of classic variant, and 1 case of columnar cells variant of PTC.

Patients with PHPT and thyroid cancer were older than patients without thyroid cancer and PHPT (62±9.5* versus* 52±15.8; p=0.03). There were no differences between the patients with and without DTC according to gender, serum calcium, phosphorus, vitamin D3 levels, urinary calcium and phosphorus, or creatinine clearance (Table 2). Serum iPTH levels were higher in patients with DTC, 5 without statistical significance (p=0.10); however, in the logistic regression model, that included age, PTH, gender, serum calcium, and vitamin D, they showed a significantly association between preoperative PTH levels and the presence of DTC (p=0.03), with an estimated odds ratio of 5.16 (95% confidence interval [CI] 1.08-24.7) (Table 3).

## 4. Discussion

We present our consecutive series of patients with symptomatic PHPT in a third level attention center. We found a high prevalence of PTC, more frequent in woman patients of old age. All patients with PTC presented higher presurgery iPTH levels; higher preoperative levels of iPTH were associated with PTC (p= 0.03) [OR 5.16 (95% CI: 1.08-24.7)]. We found no difference in calcium, phosphorus, and vitamin D in both groups.

PHPT is the principal cause of hypercalcemia and one of the endocrine diseases most common [1–3]; it is more common in women and it increases with age. The subclinic or asymptomatic presentation is the most common in occidental countries like USA and Europe and its diagnosis is frequently made in an incidental way [2–5]. The follow-up studies of asymptomatic PHPT have demonstrated that it is a disease that is often progressive; after 10 to 15 years, a progression of the disease has been seen [6].

Thyroid nodules have a high prevalence in general population (18-68%), with higher incidence in women and elders. The clinical importance of thyroid nodules is the necessity of excluding thyroid cancer. PTC occurs in 7 to 15% of cases and is related to risk factors as age, sex, radiation exposition, family history, among others [13]. It calls to attention that, in patients with PHPT, the prevalence of thyroid nodular disease is similar that in general population; nevertheless, CPT is more frequent than in general population [15, 16]. The high concurrence of these two diseases in the same patients may not be a coincidence; a lot of factors may connect PHTP with CPT.

The majority of the reported studies were made in patients with PHPT in different stages of evolution [15, 16]. In the present study, in which we included patients with symptomatic PHPT, we found a prevalence of CPT in 20%. Our results show that all patients had target organ damage (osteoporosis or recurrent lithiasis) and were considered by us as long-lasting evolution.

Xue Y et al. found that, from 155 patients with PHPT, 7.7% were ill with concomitant PTC; they found lower calcium concentrations as predictor of nonmedullary thyroid cancer in patients with PHPT [16, 17]. Gul K et al. found that, from 60 patients with PHPT, 9 patients (15%) had PTC in a concomitant; they recommend the evaluation of the thyroid with ultrasonography and FNAB [17]. Milas M et al. found 55 cases of thyroid cancer (4.6%) of 1200 consecutive PHT patients; they highlighted the necessity of ultrasound performed by surgeons or endocrinologists preoperatively and preparation for possible simultaneous parathyroid-thyroid surgery [18].

Our results have found the association of PTC with high concentrations of PTH in patients who clinically have a long time of evolution of their PHPT.

Observational and experimental studies of PTH find a favorable effect of cell proliferation. It has been seen an association between high levels of iPTH and phagocytosis alteration, T cells sensibility, and B cells function, which explains immune dysfunction and a higher risk for cancer [19, 20]. It has also been seen that thyrocytes express insulin like growth factor 1 receptors (IGF-1) and produce IGF-1 in vivo. This characteristic supports the hypothesis that IGF-1 may regulate the thyroid growth and, therefore, may be involved in the pathogenesis of multinodular goiter [21]. Some authors have postulated that iPTH may stimulate hepatic synthesis of IGF-1, epidermal growth factor, and that it could increase the thyroid cellular proliferation. It has also been demonstrated that some cancers express PTH/PTHrp and are related to mitogenic signals [22]. The PTH also stimulates the renal synthesis of 1,25-dihydroxyvitamin D. The deficiency of vitamin D produces secondary hyperparathyroidism. PTH in cellular levels has also been demonstrated to increase the cellular proliferation in bone marrow and liver in vivo and T lymphocytes in vitro [22, 23].

The tumorigenic action of PTH excess may act without opposition because of vitamin D deficiency, which is frequently presented in patients with PHPT [22, 23], and contribute to the increase of tumors formation like in patients in dialysis for terminal renal disease [24]. It is known than vitamin D deficiency in patients of PHPT is associated with more sever PHPT clinical presentation, more target organ damage, and the postsurgery presentation of hungry bone syndrome [25]. In addition to its main role in calcium homeostasis, vitamin D can directly or indirectly regulate multiple signaling pathways involved in cellular proliferation, differentiation, apoptosis, inflammation, invasion, angiogenesis, and metastasis [26]. Although there is a list of cancers showing relation to low levels of vitamin D, the most prominently addressed in research so far are cancers of the breast, colon, and prostate [27]. In our series, all our patients with severe PHPT showed low concentrations of vitamin D. It is very probable that vitamin D deficiency has a permissive or additive function with other oncogenic factors in the context of symptomatic PHPT.

Other risk factors have been proposed for the coexistence of PTC and PHPT like radiation exposition, the goitrogenic effect of prolonged hypercalcemia, calcitonin elevation, and genetic factors [11, 28].

The limitations of our study are the number of patients that is limited to a single center; although all cases have white organ damage, it has been impossible to determine the time of evolution of PHPT. Even though the histopathological characteristics suggest a more aggressive behavior of the CPT, preoperative molecular markers determination is not made in a rutinary way in patients with indeterminate FNA cytology.

## 5. Conclusion

PTC is frequent in patients with symptomatic PHPT. Thyroid nodules in patients with symptomatic PHPT must be studied before parathyroidectomy. In symptomatic PHPT, higher level concentration of parathormone (PTH) was associated with higher risk of DTC.

## Figures and Tables

**Table 1:** Clinical biochemical and pathology finding in patients with severe primary hyperparathyroidism.

**Parameter**	**N=59**
Age (year)	54 ± 15

Female gender	46 (78%)

Serum Calcium (mg/dl)	11.4 (11-12-1)

Serum Phosphorus (mg/dl)	2.6 (2.2-2.8)

PTHi (pg/ml)	147 (117-243)

Vitamin D (ng/ml)	14.9± 5.36

Urinary calcium (mg/24-hour)	318 (210-373)

Creatinine clearance (ml/min)	79.4± 27.4

Hypertensive crisis	5 (8.5%)

Renal stone	28 (47%)

Osteoporosis	29 (49%)

Pancreatitis	2 (3.4%)

Hypertension	33 (56%)

Prediabetes	24 (41%)

Diabetes	6 (10%)

Histopathologic finding (%)	
Adenoma	51 (86%)
Hyperplasia	8 (14%)

**Table 2:** Characteristics of patients with severe primary hyperparathyroidism, with and without thyroid cancer.

	**With thyroid cancer**	**Without thyroid cancer**	**P**
	**n= 12**	**n=47**	
Age	62 ± 9.5	52±15.8	0.03

Female gender	11 (92%)	35 (76%)	0.20

Autoimmune thyroid disease	4 (33%)	7 (15%)	0.10

Calcium (mg/dl)	11.8 (10.9-12.5)	11.4 (11-12.1)	0.90

Phosphorus (mg/dl)	2.6± 0.4	2.5±0.4	0.20

PTHi (pg/ml)	224 (130-460)	144 (115-227)	0.10

Vitamin D (ng/ml)	15± 5.4	14.9± 5.3	0.20

Urinary calcium in 24 hours (mg/24-hour)	318 (245-345)	319(210-400)	0.78

Creatinine clearance (ml/min)	73±20.9	81.7±28.6	0.20

**Table 3:** Multivariate analysis of features potentially associated with thyroid cancer.

**Variable**	**OR**	**95**%** Confidence interval**	**P**
Age	1.05	0.98-1.12	0.11

**PTH **(pg/ml)	**5.16**	**1.08-24.7**	**0.03**

Gender	3.68	0.32-41.6	0.29

Serum calcium mg/dl	0.79	0.41-1.54	0.50

Vitamin D (ng/ml)	1.03	0.89-1.19	0.63

## Data Availability

All data generated or analyzed during this study are included in this published article. The database generated during the current study is available with the corresponding author on reasonable request.

## Abbreviations

PHPT: Primary hyperparathyroidism
HPT: Hyperparathyroidism
PTH: Parathyroid hormone
US: Ultrasound
DTC: Differentiated thyroid cancer
PTC: Papillary Thyroid Carcinoma
FNAB: Fine needle aspiration biopsy
25(OH)D3: 25-Hydroxyvitamin D or cholecalciferol
1,25(OH)2D3: 1,25-Dihydroxycholecalciferol.
